# Erratum to: Analysis of 3-dimensional interventricular septum and abnormal muscle bundles models for septal myectomy [Interact CardioVasc Thorac Surg 2021; https://doi.org/10.1093/icvts/ivab213]

**DOI:** 10.1093/icvts/ivac093

**Published:** 2022-04-11

**Authors:** Uladzimir Andrushchuk, Artsem Niavyhlas, Vitali Adzintsou, Iryna Haidzel, Hanna Model, Aliaksandr Shket

**Affiliations:** Department of cardiac surgery, Republican Scientific and Practical Centre ‘Cardiology’, Minsk, Belarus

In the originally published version of this manuscript, there were errors within Table 4. In the line for variable “Alfieri stitch” the value should read: “3 (2.9)” instead of “3 (13.3)”. For variable “Posterior semi-annulus suture plasty” should read: “8 (7.8)” instead of “8 (6.7)”. These errors are now corrected in online.

